# DAILY EXPERIENCES OF AGING: A COUNTRY-INFORMED PERSPECTIVE FROM THE SUBJECTIVE AGES PROJECT

**DOI:** 10.1093/geroni/igae098.1733

**Published:** 2024-12-31

**Authors:** Shevaun Neupert, Anna Kornadt

**Affiliations:** Chair; Co-Chair

## Abstract

The proposed symposium provides a global perspective regarding antecedents, correlates, and consequences of views on aging based on the Subjective AGES (Aging within Global everyday Ecological Studies) consortium. This culture-informed approach highlights the contextual and dynamic influences on views on aging across different temporal perspectives. We enrich the existing body of knowledge by including a broad variety of cultures and investigating how views on aging connect to daily indicators of health and well-being. First, Wu and Fung investigate the moderating role of future self views on the relationship between self continuity and preparation for old age in six cultures. Taking into account the historic context of inflation in Turkiye, Can and Neupert find that daily subjective economic status and daily subjective age are each uniquely related to daily happiness. Wirth and Rothermund show that the daily experience of expectations regarding aging in Germany influences affective experience. Finnigan and Tse show that daily flow experiences in a sample from the UK were related to younger subjective age, which in turn increased positive affect. Finally, Shrira and colleagues compare the daily coupling of ageist attitudes and negative affect in five different countries using an integrated data analysis approach. Our findings shed light on country-specific and global relationships between views on aging and development. We showcase how experiences of aging are shaped by contextual and dynamic influences.

